# The Practice of Physical Activity on Psychological, Mental, Physical, and Social Wellbeing for Breast-Cancer Survivors: An Umbrella Review

**DOI:** 10.3390/ijerph191610391

**Published:** 2022-08-20

**Authors:** Marta Zanghì, Luca Petrigna, Grazia Maugeri, Velia D’Agata, Giuseppe Musumeci

**Affiliations:** 1 Department of Biomedical and Biotechnological Sciences, Anatomy, Histology and Movement Science Section, School of Medicine, University of Catania, Via S. Sofia n°87, 95123 Catania, Italy; 2 Research Center on Motor Activities (CRAM), University of Catania, Via S. Sofia n°97, 95123 Catania, Italy

**Keywords:** tumor, exercise, movement, exercise training

## Abstract

(1) Background: The number of breast-cancer patients and survivors is increasing in the last years. Physical activity seems to be a feasible and useful complementary intervention to improve the physical, psychological, and social spheres and decrease some symptoms, especially for survivors. Consequently, the objective of the present umbrella review was to analyze the efficacy of different physical-activity interventions in the physical, mental, and social spheres of breast-cancer survivors. (2) Methods: Systematic reviews and meta-analyses of randomized controlled trials on breast-cancer survivors and physical-activity effects were searched on the electronic databases PubMed, Web of Science, and Scopus till 9 August 2022. The quality of the studies included was evaluated, and the results were narratively analyzed. (3) Results: Physical-activity intervention generally improves the physical, mental, and social spheres of breast-cancer survivors, but the studies included present heterogeneity in the protocols adopted. (4) Conclusions: A well-structured and planned physical-activity intervention is useful for improvements in the physical, mental, and social spheres of breast-cancer survivors, but the studies presented high heterogeneity. Yoga seems to be the most effective physical intervention to complement medical therapy.

## 1. Introduction

New treatment protocols, intervention techniques, and early diagnosis increase the number of breast-cancer (BC) survivors, as well as the possibility of reaching an older age; however, they also cause BC survivors to present other diseases and comorbidity situations [1]. Furthermore, in old age, there are profound changes in body composition, which could generate sarcopenia, with decreases in strength and the aerobic and functional capacities, and an increase in the body-fat volume [2]. A second aspect to consider is that BC patients and survivors frequently experience higher levels of anxiety and depression, poorer quality of life (QoL), higher levels of fatigue, poorer physical functioning, and urinary dysfunction [3]. A final aspect to consider is the limited understanding of cancer in the social domains, and especially in social functioning [4]. These coexisting medical conditions could increase the risk of treatment toxicity [5] and, consequently, the risk of death.

The World Health Organization defined health as complete physical, mental, and social wellbeing [6], highlighting the attention on these three elements. Furthermore, programs adequately designed and adapted to the patient, when compared with medical intervention or treatment, reduce health risks, and limit the healthcare-system costs [7,8]. Usually, a well-planned intervention brings about positive outcomes from a physical point of view [9], decreases cardiovascular disease, hypertension, type 2 diabetes, site-specific cancers, and mental-health problems (such as anxiety and depression), reduces adiposity, and improves cognitive health and sleep quality [10].

The regular practice of a physical activity, always alongside medical therapy, is also essential for the prevention and treatment of BC. Physical-activity programs during illness reduce the risk of BC-related death after diagnosis [11,12]. It seems that being physically active throughout life prevents BC [13] and reduces the BC incidence [14], mortality, and morbidity [15,16], and especially if associated with weight loss [17] and among postmenopausal women [18]. It is also fundamental to be physically active before the diagnosis; indeed, there is an inverse relationship between the physical-activity level and all-cause and BC-related deaths and events [11,12]. These aspects make it interesting to better understand physical activity’s role in BC survivors, and especially because physical activity is a safe, feasible, and efficacious intervention in BC patients who are undergoing different types of treatment [19]. It is also useful in the reduction in or management of the symptoms [3]. Physical activity could therefore help reintegrate patients into daily routines, work, and family life [20]. Exercise training during BC treatment is often associated with a progressive improvement in physical, physiological, and psychological health, as well as functional parameters [1,21]. It creates a blend directly with the tumor and its microenvironment, which may promote its use as an additional coadjuvant therapy [22]: a long-term physical-activity intervention reduces the risk of BC through increased global DNA methylation [23].

Different physical-exercise interventions at different durations adapted to the physical conditions of the person seem to reduce the risk of BC [24]. Aerobic training, resistance training [25], interventions based on flexibility, yoga, tai chi, qigong, and Pilates [26], or exercise in general [27], have been proposed, with different outcomes and conclusions. Despite the large number of studies on this topic, heterogeneity in the results and methodological limitations have made the findings of the studies limited [28]. This confusion on the effects of physical-activity interventions on BC symptoms makes it necessary to deeply investigate the topic [29] to also understand the magnitude of the positive effects of physical activity among BC survivors [30]. Consequently, the objective of the present study was to evaluate previously published systematic reviews and meta-analyses of randomized controlled trials with the same topic [31]. The question analyzed was the study of the effects of physical activity on BC survivors, and we extrapolated information about exercise-training effects on BC-related symptoms, focalizing the attention on the physical, mental, and social spheres.

## 2. Materials and Methods

This umbrella review followed the Preferred Reporting Items for Systematic Reviews and Meta-Analyses (PRISMA) guidelines [32]. PRISMA checklist [33] and the search strategy is included in Supplementary Materials.

### 2.1. Search Strategy

The included articles were searched on the electronic databases PubMed, Web of Science, and Scopus. Systematic reviews and meta-analyses were included if they were published up to the 09th of August 2022. Different keywords were adopted: (a) breast cancer and breast neoplasm; (b) exercise and physical activity; (c) review and meta-analysis. The keywords were matched through the Boolean operators AND or OR. A string was adopted: (“breast cancer” OR “breast neoplasm”) AND (exercise OR “physical activity”) AND (review OR meta-analysis), in the three databases.

### 2.2. Eligibility Criteria

Inclusion and exclusion criteria for population, intervention, comparison, outcomes, and study design (PICO-S) were considered. The population was composed of BC survivors (despite the age). Reviews were excluded if the sample investigated included other types of cancer diagnoses. Studies were excluded if the physical-exercise intervention was not structured. The intervention had to include structured physical exercise. The comparison was with control groups, both sedentary and active, and pre-and postintervention. Outcomes had to be parameters related to the physical, mental, and social characteristics. Other studies that were designed differently from systematic reviews and meta-analyses of randomized controlled trials were excluded. Only English-written studies were included.

### 2.3. Data Sources, Study Sections, and Data Extraction

In the first step, manuscripts were stored in EndNote X8 (EndNote version X8; Thompson Reuters, New York, NY, USA), and the duplicate selection was performed. In the second phase, two independent investigators screened the reviews against the eligibility criteria based on the title, abstract, and full text. The eventual disagreement between the two investigators was solved by the principal investigator.

Information related to the first author and year of publication, review methodology, databases screened, number of reviews included, objective of the study, risk of bias assessment and score, conclusion of the study, population screened, training characteristics, and main results were stored in the tables. A descriptive and narrative synthesis was adopted to analyze the results. A meta-analysis was not performed due to the possibility that including studies considered in more than one systematic review increased the risk of bias [34].

### 2.4. Quality Assessment

The quality of the included systematic reviews and meta-analyses of randomized controlled trials was assessed with the rating scale: “Assessment of Multiple Systematic Reviews” (AMSTAR) [35]. It consists of 11 items and presents the reliability and validity [36]. Studies with a final score between 0 and 4 were considered of poor quality. A score between 5 and 7 was considered of moderate quality. A score above 8 indicated high quality. A score of 0 was adopted if “no sufficient information were available”, and a score of 1 was adopted if “enough information” was collected. All included reviews were scored independently by two investigators, and disagreement was resolved by the principal investigator.

## 3. Results

**Figure 1:**
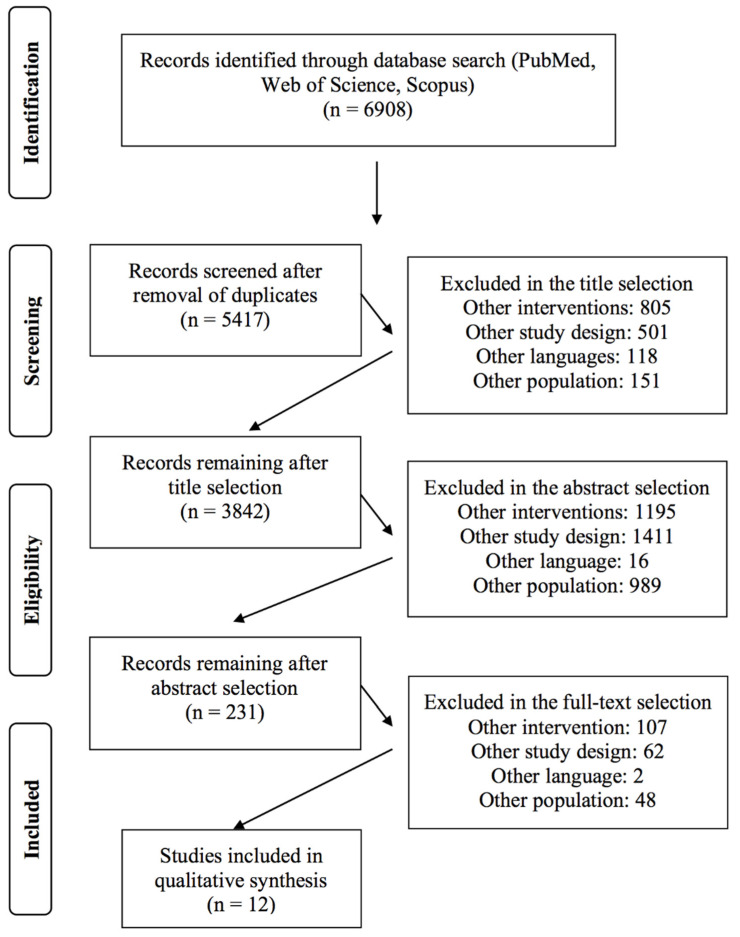
Flow chart of the selection criteria process of the included studies.

A total of 6908 studies (PubMed: 1310; Web of Science: 2835; Scopus: 2763) were found after the search of the electronic databases. After duplicate removal, title, and abstract screening, a total of 253 studies were collected for full-text analysis. A final number of 12 systematic reviews and meta-analyses are included in this umbrella review. The screening process is summarized in Figure 1.

### 3.1. Characteristics of the Included Studies

Nine studies adopted PRISMA guidelines, and one study also adopted the Cochrane Handbook. MEDLINE (Pubmed), Scopus, Web of Science, PsycINFO, CENTRAL, and EBSCO were mainly adopted to search the articles. The number of included reviews in the studies ranged from eight to fifty-seven. Four studies adopted the Cochrane Handbook to detect the quality of the included studies, while three studies adopted the Physiotherapy Evidence Database (PEDro) scale. More details about the study characteristics are provided in Table 1.

### 3.2. Physical, Psychological, and Social Sphere Outcomes

The participants in the included interventions were not only BC survivors, but there were also patients at stages 0 to 4 undergoing therapy and surgery. The age ranged from 18 to 83 years, but most of the studies had an age that ranged from 45 to 55 years. Two studies had no information in this regard [41,43].

The interventions adopted were yoga (n = 3), tai chi (n = 2), aerobic training, and resistance training (n = 4), or a combination of both training methods (n = 2). Water exercise and horseback riding were adopted in one study, such as stretching exercises. The proposed exercise training ranged from 6 weeks to 52 weeks, with the majority of the interventions being 18 weeks long. The weekly frequency ranged from 1 to 6 times (mean: 3 times a week), and the duration of the single session was from 30 to 120 min (mean: 90 min).

Aerobic training statistically significantly reduced cancer-related fatigue, while aerobic-resistance training seemed to result in the greatest benefit in terms of physical functioning [41]. Another of the included studies detected that walking at a high intensity had the largest effect on the cardiac function [37]. The study of Ramirez-Velez, 2021 [46], detected that exercise training reduced cancer-related fatigue, with a statistical difference. Exercise training decreased the systolic and diastolic blood pressure, triglyceride levels, and body-mass index; with a statistical difference, it increased the peak oxygen uptake, maximal oxygen consumption, and high-density leptin cholesterol, while no statistical differences were detected in the changes in the mean arterial pressure, peak heart rate, and peak respiratory exchange ratio [47]. Supervised exercise training statistically improves functional and physical wellbeing [45]. Yoga presented statistically significant effects on cancer-related fatigue [39]. Tai chi and conventional therapy are more effective for cancer-related fatigue at 3 months [44]. Tai chi also improved cancer-related fatigue, with no statistical difference in the bone mineral density, body-mass index, or muscle strength [48]. The positive effects of exercise on cancer-related fatigue are also confirmed by other studies [43].

Yoga, if compared with the treatment group, had small short-term effects on the QoL and functional, social, and spiritual wellbeing, but when compared with an active control, large short-term effects on mental wellbeing, anxiety, depression, stress, and psychological distress were detected [38]. Yoga also presented statistically significant effects on anxiety and depression, with an overall enhancement of QoL [39]. The study of Liu, 2020 [44], found a statistically significant improvement after tai chi in QoL at 3 months. Tai chi and conventional therapy are more effective on QoL at 3 and 6 months. There were no statistical differences from conventional therapy in improving the sleep parameters, depression, or body-mass index. Tai chi also improved emotional wellbeing, with a statistical difference [48]. The study by Ramirez-Velez, 2021 [46], detected that exercise training reduced, with a statistical difference, anxiety and depression, and produced increases in body image, QoL, and emotional function. 

Supervised exercise training has nonstatistically significant effects on the social and emotional wellbeing domains [45]. There is a significant heterogeneity depending on whether the intervention is proposed in groups or not [40]. More details of the studies are included in Table 2.

### 3.3. Risk of Bias Assessment

The quality of the included studies ranged from four to ten, with a mean of 4/9. Within the included studies, the overall quality is mainly moderate, and the risk of bias is medium. Two studies had no scores. The results of one review are unclear. A summary is provided in Table 1 and Table 3.

## 4. Discussion

Systematic reviews and meta-analyses of randomized controlled trials are highly heterogeneous. Despite this important limitation, the findings suggest that exercise training, regardless of its structure and typology, has positive outcomes on the physical, psychological, and social health spheres in BC survivors. Among the activities analyzed, yoga was ideal because its effects are on the three spheres analyzed. It is fundamental to consider that exercise training can only complement medical therapy and not replace it.

Most of the physical-training interventions of the included studies, even if there were differences in the typology (aerobic, resistance training, or combining both), structure (duration, frequency, intensity), or modality (biking, swimming walking, riding a horse), presented improvements in physical functioning [41] and cardiovascular-system functions [47]. These findings suggest the importance of practicing physical activity to reduce the risk of cardiovascular problems and improve the overall QoL. The results of physical activity are also indirect; indeed, one of the included studies [42] correlated the reduction in body weight with the reduction in the fasting insulin levels. Our findings are similar to other studies in which similar positive physical outcomes were detected [49,50].

From a psychological point of view, exercise training improves psychological health [38] anxiety and depression, QoL, and emotional function [39,46]. Mindful physical activities, such as yoga, are suggested for their positive outcomes, and especially on mental health (psychological and social health) [38,39]. Our findings are in line with other studies in which the positive effects of physical activity on the psychological outcomes and QoL in patients after treatment for BC were detected [51]. In a few words, exercise training has a positive effect on the health outcomes in cancer survivors [52]. Another adopted “mind-based” intervention was tai chi, but the results are contradictory, with one study suggesting improvements in cancer-related fatigue [44], while another study asserts that there is a lack of a statistically significant improvement in the QoL and other clinical endpoints [48]. Two studies detected no long-term improvements in mental wellbeing, physical wellbeing, anxiety, depression, psychological distress [38], and QoL improvement [48], which makes it necessary to consider physical activity as a lifestyle and not a temporary intervention. One deeply investigated aspect was the QoL, and different studies [39,45,46] detected its improvement after exercise training; this trend was also observed in the literature [49,52]. Different scientific articles detected positive outcomes of exercise training (despite the training-intervention modality) on cancer-related-fatigue symptoms [39,41,43,46], and especially when proposed with conventional supportive care interventions [44]. The cancer-related-fatigue parameters were reduced, and especially with aerobic training [41,53]. These findings, also supported by the literature [52], and especially if the training is supervised [53], have to be seriously considered to, first, allow BC patients the practice of physical exercise, and, second, increase the possibility of being more active in daily living.

From a social point of view, the reviews of the literature, again, suggest that yoga [38,39] and tai chi (with conventional support care) [44] have positive outcomes. Another aspect to consider is that, even if no statistically significant effects were detected [40], it is fundamental to work in groups to improve the social aspects and interpersonal relationships, and, as the literature suggests, this has also positive outcomes on social issues [54].

The strength of this study is that it provides community feedback about the positive outcomes of physical activity on BC survivors in terms of health. Fundamentally, clinicians consider this aspect after the critical phase and during recovery, and especially because, despite the typology of physical exercise, postdiagnosis physical activity is associated with an increase in the survival rate among BC survivors [55]. It is also important for BC patients to be active to decrease the mortality risk [56]. Physical activity, whether prediagnosis or postdiagnosis, is associated with a better prognosis for BC [57].

The most important limitation of this review is the heterogeneity of the included studies in the methodology (eligibility criteria, outcomes measures) and the sample included (age, cancer trajectory). Heterogeneity was also detected in the intervention characteristics in terms of duration and frequency. This limitation made it difficult to synthesize the data. In our manuscript, we tried to include only moderate- or high-quality systematic reviews and meta-analyses, but the included original articles presented different results in the quality assessment. Furthermore, as we detected heterogeneity between the reviews investigated, heterogeneity was also within the articles included in the reviews. Future studies should focalize attention on the different effects of physical activity on BC patients under therapy or under other conditions.

## 5. Conclusions

The benefits that arise from this practice of different typologies of exercise training (the studies included were highly heterogeneous) are the physical, psychological, and social health of BC survivors. Among the interventions proposed in the included studies, yoga seems to be an appropriate exercise because it has positive outcomes in the physical, psychological, and social spheres. Systematic reviews and meta-analyses of randomized controlled trials over the last ten years affirm that, despite the cancer history, exercise training is suggested.

## Figures and Tables

**Table 1:** Characteristics of the included studies.

First Author, Year	Guideline	Databases Searched	Main Objective	N of Studies	Risk of Bias	Main Conclusions
Bluethmann, 2015 [37]	PRISMA	Medline; PsycINFO; Ebsco…	ET on behavior change and characteristics of interventions	14	NI	Effectiveness in short-term behavior changes of ET but varied based on intervention and intensity of supervision/monitoring.
Cramer, 2012 [38]	PRISMA	MEDLINE, Cochrane Library, PsycInfo, EMBASE…	Yoga on health-related QoL and psychological health	12	Cochrane tool: unclear	Evidence for short-term effects of yoga in improving psychological and social health.
El-Hashimi, 2019 [39]	NI	CINAHL, CENTRAL, MEDLINE, PsycINFO, Scopus, SPORTDiscus...	Yoga on QoL compared with other physical intervention	8	NI	Interventions in general (not yoga-specific) diminished CRF, anxiety, depression, overall enhancement of QoL, and in the social sphere.
Floyd, 2009 [40]	NI	PsycINFO, CINAHL Plus, MEDLINE, Cochrane	Group as compared to individual ET would show greater improvement in QoL	17	NI	Group exercise interventions showed no advantage.
Juvet, 2017 [41]	PRISMA	Cochrane Library, MEDLINE, EMBASE, PsycINFO, PEDro…	ET on QoL. Focus on self-reported physical functioning and CRF	25	Norwegian KnowledgeCentre for the Health Services: moderate	ET program can produce short-term improvements in physical functioning and can reduce CRF.
Kang, 2017 [42]	PRISMA and Cochrane	PubMed, EMBASE, CENTRAL, CINAHL, SportDiscuss…	ET on insulin markers	18	Cochrane tool: low	Exercise reduces fasting insulin levels. This may be due to exercise-induced reductions in body weight.
Lin, 2021 [43]	PRISMA	Cochrane Library, EMBASE, Medline, CINAHL, PsycINFO…	ET type, duration, and intensity on CRF	9	JBI-MASTARI: high	ET can reduce CRF.
Liu, 2020 [44]	PRISMA	OVID MEDLINE, AMED, EMBASE, CINAHL, CENTRAL…	Effectiveness of tai chi on CRF	16	PEDro score:moderate to high	Tai chi does not improve CRF. It significantly relieves CRF symptoms and social health when used with conventional support care.
Meneses-Echávez, 2015 [45]	PRISMA	MEDLINE, EMBASE, Scopus, CENTRAL, CINAHL	Pooled effects of supervised ET on CRF	9	PEDro score:low	Supervised exercise reduces CRF. High volumes are also safe and effective on QoL.
Ramírez-Vélez, 2021 [46]	PRISMA	MEDLINE, Embase, Web of Science	ET (type, intensity, volume, and frequency) on mental wellbeing	57	PEDro: medium	ET significantly reduced anxiety, depression, and CRF, and there were increases in body image and QoL and emotional function.
Wang, 2021 [47]	PRISMA	MEDLINE, CINAHL, Cochrane Library, Web of Science and Scopus…	ET on the cardiovascular system during the convalescence	11	Cochrane tool: low	ET could improve the associated cardiovascular-system function.
Yan, 2014 [48]	NI	MEDLINE, EMBASE, CINAHL…	Tai chi on QoL and other clinical outcomes	5	Cochrane tool: low	Lack of sufficient evidence to support tai chi to improve QoL/other clinical endpoints.

Note: CRF: cancer-related fatigue; CENTRAL: Cochrane Central Register of Controlled Trials; ET: exercise training; NI: no information; PRISMA: Preferred Reporting Items for Systematic Reviews and Meta-Analyses; QoL: quality of life; RT: resistance training; SCIELO: Scientific Electronic Library On-Line.

**Table 2:** Characteristics of the interventions.

First Author, Year	Participants	Intervention	Interventions Characteristics (L; F; D)
Bluethmann, 2015 [37]	Survivors; stages I, II, and IV, undergoing therapy and surgery	Walking, group exercise, HB (mix)	Mean: 17 weeks; 3 times weekly; 45 min or less
Cramer, 2012 [38]	Stages 0–4; survivor; with active treatment	Different yoga types	1–24 weeks; 1–7 times weekly; 45–120 min
El-Hashimi, 2019 [39]	Nonmetastasized; the first course of cancer treatment; survivors	Different yoga types. Personalized, small group, DVDs, leaflets.	4–12 weeks (median: 10); 1–3 times weekly (median: 1); 60–90 min
Floyd, 2009 [40]	Primarily stage I and/or stage II	Range of exercise types	14 weeks; 3 times weekly; 45 min
Juvet, 2017 [41]	Early-stage; surgical followed by therapy	AT, RT, AT–RT	NI
Lin, 2021 [43]	Survivors, stages 0–3, completed treatments, no hormonal therapy	Yoga, mixed AT, water exercise, horseback riding, cycle ergometers	NI
Liu, 2020 [44]	Active treatment	Tai chi	10–24 weeks; 1–4 times weekly; 30–120 min
Meneses-Echávez, 2015 [45]	Survivors	AT, RT, stretching exercises	21 weeks (SD: 16); 2.5 times (SD: 0.7) weekly; 44 min (SD: 15)
Ramirez-Velez, 2021 [46]	Survivors; all stages, undergoing therapy	AT, RT, AT–RT program combining AT, strength, and flexibility. Supervised, HB, mix.	6–52 weeks (mean: 18); 3 times weekly (range: 1–6)
Wang, 2021 [47]	Completed the primary treatment	AT, RT, functional training	8–24 weeks (mean: 16); 3–5 times weekly
Yan, 2014 [48]	Diagnosed breast cancer	Tai chi	10–24 weeks; 40–90 min

Note: AT: aerobic training; D: duration; F: frequency; HB: home-based; L: length; RT: resistance training; SD: standard deviation.

**Table 3:** Quality assessment through the “assessment of multiple systematic reviews” (AMSTAR) of the included systematic reviews.

First Author, Year	1	2	3	4	5	6	7	8	9	10	11	Tot
Bluethmann, 2015 [37]	0	1	0	0	0	1	0	0	1	0	1	4
Cramer, 2012 [38]	0	1	1	0	0	1	1	0	0	1	0	5
El-Hashimi, 2019 [39]	0	1	1	0	0	1	1	0	1	0	1	6
Juvet, 2017 [41]	0	1	1	1	0	1	1	1	1	0	1	8
Lin, 2021 [43]	0	1	1	0	0	1	1	0	1	0	0	5
Liu, 2020 [44]	0	1	1	0	0	1	1	1	1	0	1	7
Meneses-Echávez, 2015 [45]	1	1	1	0	0	1	1	1	1	1	1	8
Ramírez-Vélez, 2021 [46]	0	1	1	0	0	1	1	1	1	0	1	7
Wang, 2021 [47]	1	1	1	0	0	1	1	1	1	1	1	9
Yan, 2014 [48]	0	1	1	0	0	1	1	0	1	0	0	5

1. Was an “a priori” design provided? 2. Was there duplicate study selection and data extraction? 3. Was a comprehensive literature search performed? At least two electronic sources include years and databases used (e.g., Central, EMBASE, and MEDLINE) 4. Was the status of publication (i.e., grey literature) used as an inclusion criterion? 5. Was a list of studies (included and excluded) provided? 6. Were the characteristics of the included studies provided? 7. Was the scientific quality of the included studies assessed and documented? 8. Was the scientific quality of the included studies used appropriately in formulating conclusions? 9. Were the methods used to combine the findings of studies appropriate? 10. Was the likelihood of publication bias assessed? 11. Were potential conflicts of interest included?

## Data Availability

Not applicable.

## Supplementary Materials

The following supporting information can be downloaded at: https://www.mdpi.com/article/10.3390/ijerph191610391/s1, PRISMA checklist and the search strategy is included.
